# Effects of Air Pollution Exposure during Preconception and Pregnancy on Gestational Diabetes Mellitus

**DOI:** 10.3390/toxics11090728

**Published:** 2023-08-24

**Authors:** Lei Cao, Ruiping Diao, Xuefeng Shi, Lu Cao, Zerui Gong, Xupeng Zhang, Xiaohan Yan, Ting Wang, Hongjun Mao

**Affiliations:** 1 China Institute for Radiation Protection, Taiyuan 030006, China; 2 Tianjin Key Laboratory of Urban Transport Emission Research & State Environmental Protection Key, Laboratory of Urban Ambient Air Particulate Matter Pollution Prevention and Control, College of Environmental Science and Engineering, Nankai University, Tianjin 300071, China; 3 Handan Maternal and Children Health Hospital, Handan 056001, China

**Keywords:** air pollution, gestational diabetes mellitus, cohort study, preconception, pregnancy

## Abstract

This study aimed to investigate the association between air pollution and gestational diabetes mellitus (GDM) in small- and medium-sized cities, identify sensitive periods and major pollutants, and explore the effects of air pollution on different populations. A total of 9820 women who delivered in Handan Maternal and Child Health Hospital in the Hebei Province from February 2018 to July 2020 were included in the study. Logistic regression and principal component logistic regression models were used to assess the effects of air pollution exposure during preconception and pregnancy on GDM risk and the differences in the effects across populations. The results suggested that each 20 μg/m^3^ increase in PM_2.5_ and PM_10_ exposure during preconception and pregnancy significantly increased the risk of GDM, and a 10 μg/m^3^ increase in NO_2_ exposure during pregnancy was also associated with the risk of GDM. In a subgroup analysis, pregnant women aged 30–35 years, nulliparous women, and those with less than a bachelor’s education were the most sensitive groups. This study provides evidence for an association between air pollution and the prevalence of GDM, with PM_2.5_, PM_10_, and NO_2_ as risk factors for GDM.

## 1. Introduction

Air pollution is a dangerous environmental risk factor contributing to the global burden of disease [1]. Evidence of the adverse health effects of air pollution has increased significantly over the past decade. As a specific population group, pregnant women and their babies bear a greater burden of disease due to air pollution [2]. To adapt to changes in metabolism and oxygen consumption during pregnancy, the physiological characteristics of pregnant women undergo significant short-term changes, including increased alveolar ventilation, an increased blood volume, and fat deposition, which predispose them to cardiovascular disease [3]. Although the pathophysiological mechanisms between ambient air pollution and maternal disease remain unclear, several possible mechanisms have been proposed, including systemic inflammation, oxidative stress, and endothelial dysfunction [4,5]. Systemic inflammation and oxidative stress induced by air pollution can lead to insulin resistance, which is the underlying mechanism of gestational diabetes mellitus (GDM) in pregnant women [6].

GDM is a common complication of pregnancy, which is defined as hyperglycemia and hyperinsulinemia caused by a woman’s first carbohydrate intolerance during pregnancy. GDM usually develops in the second trimester and disappears after the baby is born. GDM is detrimental to the short-term and long-term health of mothers and their fetuses. For pregnant women, GDM is associated with an increased risk of perinatal complications, such as pre-eclampsia, dystocia, and caesarean sections [7,8,9]. Approximately one third of women with GDM will eventually develop type 2 diabetes, and women with GDM also have a higher long-term risk of cardiovascular disease than women without GDM [10]. For fetuses, GDM is associated with macrosomia, organic macrosomia (an abnormal enlargement of the fetal organs), shoulder dystocia, birth injury, impaired glucose tolerance, obesity, and intellectual disability [11,12,13,14,15].

Understanding the health effects of air pollution is important for reducing the burden of maternal and child disease and formulating policies for preventing and controlling air pollution. Numerous studies have investigated the association between air pollution and GDM. Some epidemiological studies have shown an adverse effect of air pollution on GDM [16,17,18,19], but other studies have not found such an association [20,21]. Conclusive epidemiological evidence on the association between exposure to air pollution and GDM is still lacking. Currently, most studies have been carried out in European and American countries with low levels of air pollution. Previous studies have shown clear ethnic differences in the incidence of GDM [22]. However, research data on non-western populations are very limited. Studies in China have been limited to large cities or urban areas [16,17,18,19,22,23,24]. However, air pollution in small- and medium-sized cities cannot be ignored, and the health of their inhabitants also deserves attention. In addition, some studies have included several correlated pollutants simultaneously in a logistic regression model to adjust for the effect of additional pollutants when examining the effect of a single pollutant [16,17,18]. This multicollinear relationship between independent variables may increase the error in parameter estimation, or even cause the positive and negative regression coefficients to be reversed, leading to a contradiction in the interpretation of the results [25,26,27].

With the rapid urbanization and industrialization around the world, especially in developing countries, the problem of air pollution is becoming increasingly serious. In recent years, China’s small- and medium-sized cities have accelerated the pace of their construction, and local governments have paid attention to economic development while neglecting environmental protection, making them more polluted than large cities [28]. As the heart of the Central Plains Economic Zone and a major transport hub in northern China, the air pollution in Handan is extremely severe [29]. According to the bulletin issued by the Ministry of Ecology and Environment from 2017 to 2020, Handan ranked in the bottom five in the list of urban ambient air quality [30,31,32,33]. The aim of this study was to re-examine the relationship between air pollution and GDM in small- and medium-sized cities with increasing pollution, identifying sensitive periods and key pollutants. Accordingly, using Handan City, Hebei Province, as an example, logistic regression models were used to assess the effects of preconception and pregnancy exposure to air pollutants, including inhalable particulate matter (PM_10_), lung-entering particulate matter (PM_2.5_), sulfur dioxide (SO_2_), nitrogen dioxide (NO_2_), carbon monoxide (CO), and ozone (O_3_). Due to the co-occurrence of several pollutants in the real environment, principal component logistic regression models were used to assess the effects of each air pollutant, adjusted for the remaining five pollutants. In addition, a subgroup analysis of pregnant women was performed to explore the differences in the effects of air pollution on different groups, in order to provide a reference for targeted public health protection.

## 2. Materials and Methods

### 2.1. Study Population and Design

**Figure 1:**
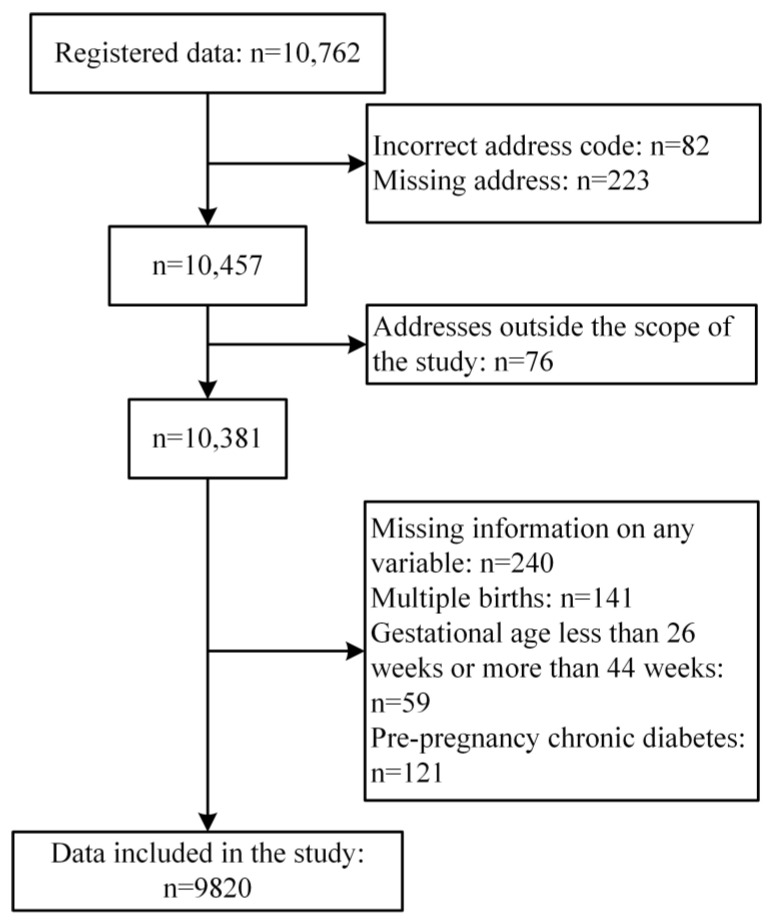
Flowchart for screening study participants.

This birth cohort study was conducted in Handan City, Hebei Province, China. Handan City is located in the southern part of the Hebei Province and has a warm, temperate, continental monsoon climate with four distinct seasons. It covers 6 districts, 11 counties, and 1 county-level city, with a total area of 12,000 km^2^. The total registered population is 10.57 million, with a permanent population of 9.55 million and a fertility rate of 11.80%. The study was a retrospective cohort study. The participants were women who gave birth at the Handan Maternal and Child Health Hospital between February 2018 and July 2020. Handan Maternal and Child Health Hospital receives more than half the deliveries of pregnant women in Handan each year, with case data covering the entire city. The hospital records detail maternal and fetal characteristics, as well as clinical data on pregnancy and delivery. Pregnant women with an address outside the Handan administrative district, missing information for any variable, multiple births, a gestational age less than 26 weeks or more than 44 weeks, and pre-pregnancy chronic diabetes were excluded from this study. Ultimately, 9820 pregnant women were included in the analysis. The screening details are shown in Figure 1. This study did not require review or approval, as it was based on a de-identified dataset.

### 2.2. Outcome and Covariates

Handan Maternal and Child Health Hospital follows the 2010 International Gestational Diabetes Association diagnostic criteria for GDM. Women were screened for GDM using an oral glucose tolerance test between 24 and 28 weeks of gestation, and GDM was diagnosed if their glucose level after a 75 g glucose load was any of the following: 0 h (fasting blood glucose) ≥ 5.1 mmol/L; 1 h ≥ 10 mmol/L; or 2 h ≥ 8.5 mmol/L. In addition, women identified as high risk may be screened early during pregnancy, with the following levels considered to be abnormal: blood glucose level of ≥11.1 mmol/L 2 h after a 75 g oral glucose load or at any time; or fasting blood glucose of ≥7.0 mmol/L.

We assessed the following potentially relevant covariates based on previous evidence [34,35,36,37,38]: maternal age (<25, 25–30, 30–35, and >35 years old), educational level (less than bachelor and bachelor or above), health insurance (none, employee medical insurance, or urban and rural medical insurance), parity (nulliparous or multiparous), conception year (2017, 2018, or 2019), conception season (spring (March–May), summer (June–August), autumn (September–November), or winter (December–February)), and previous adverse pregnancy and childbirth (yes or no).

### 2.3. Exposure Assessment

**Figure 2:**
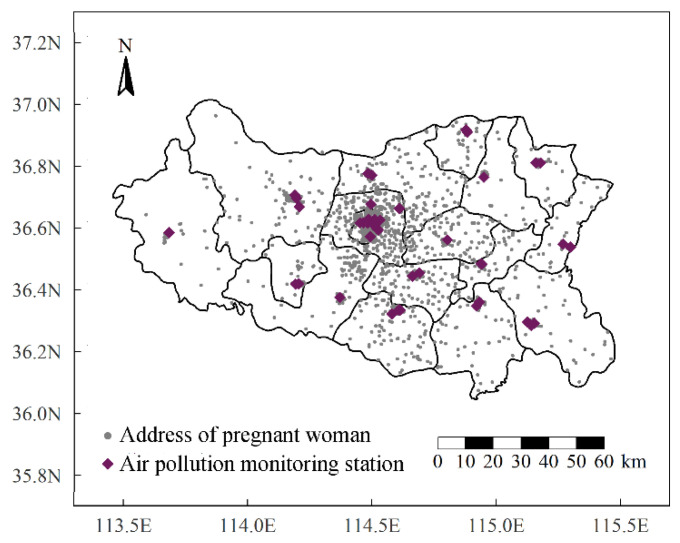
The map of location of study sites and pregnant women’s addresses and air-pollution-monitoring stations.

Data on the daily mean concentrations of six air pollutants, including PM_10_, PM_2.5_, SO_2_, NO_2_, CO, and O_3_, in Handan were obtained from the Handan Ecology and Environment Bureau at 44 monitoring sites covering all exposure windows for all the participants. The distribution of the maternal addresses and monitoring locations is shown in Figure 2. There were enough monitoring stations with a uniform spatial distribution. When a monitoring network has a sufficient spatial density, the use of direct fixed measurements avoids the potential bias associated with the use of residence-based exposure models with an uncertain validity [17,39]. Therefore, after excluding participants who were more than 20 km away from a monitoring station, the pollutant concentration data from the nearest monitoring station were directly matched to the pregnant women [40,41]. Then, for each pollutant, the average exposure concentrations were calculated in the following windows based on the gestational age and delivery date of each pregnant woman: (1) preconception (13 weeks before pregnancy, Pre_T); (2) first trimester (1–13 gestational weeks, T1); (3) second trimester (14–26 gestational weeks, T2); and (4) the first two trimesters (T). To ensure that exposure preceded outcome, exposure after 26 weeks of gestation was not included.

### 2.4. Statistical Analyses

Differences in the selected characteristics between the pregnant women with and without GDM were compared using the χ^2^ test. A descriptive analysis of the air pollution exposure levels of the pregnant women in different exposure windows was performed, and the correlation between the average exposure levels of the pollutants in different exposure windows was shown using the Spearman correlation coefficient.

Logistic regression models were used to evaluate the effect of air pollutants on GDM in each exposure window. To facilitate a discussion of the results and a comparison with other literature data, the pollutant concentrations were used as continuous variables to express the results of the study. Odds ratios (ORs) and 95% confidence intervals (95% CIs) of GDM were estimated with each 20 μg/m^3^ increase in PM_10_, 20 μg/m^3^ increase in PM_2.5_, 20 μg/m^3^ increase in O_3_, 10 μg/m^3^ increase in SO_2_, 10 μg/m^3^ increase in NO_2_, and 0.5 mg/m^3^ increase in CO during each exposure window. Two single-pollutant logistic models were fitted. Model 1 was a crude model and model 2 was adjusted for all covariates. Detailed methods are provided in Part 1 of the Supplementary Materials.

Due to the presence of multiple pollutants in the environment of the pregnant women, it was necessary to adjust for other pollutants to better estimate the effects of each pollutant. The parameter estimation for the logistic regression models required the variables to be independent of each other. However, there was a strong correlation between the six pollutants, and multicollinearity would occur if all six pollutants were entered directly and simultaneously into the model. Therefore, the principal component logistic regression method was used for the multi-pollutant model [25,26,27]. Two multi-pollutant models were fitted. Model 1 was a rough model with the first three principal component variables of the six pollutants treated as independent variables (Table S2); and model 2 was adjusted for all covariates, with the first eight principal component variables of the six pollutants and all the covariates treated as independent variables (Table S3). Detailed methods are given in Part 2 of the Supplementary Materials.

In addition, the participants were stratified by age, parity, previous adverse pregnancy and childbirth, and educational level, and subgroup analyses were performed with the single-pollutant and multi-pollutant models, respectively, adjusting for all the covariates, except the grouping variable. All the statistical analyses were performed using the R software, version 3.5.3, with a two-tailed test and an alpha level of 0.05.

## 3. Results

### 3.1. Study Population

The characteristics of the participants are shown in Table 1. Of the 9820 participants included in the analysis, approximately 3.79% of the pregnant women were diagnosed with GDM. The results of the χ^2^ test showed that there were significant differences in age and previous adverse pregnancy and childbirth between the pregnant women with and without GDM (*p* < 0.05). Pregnant women over 35 years of age were more likely to have GDM, with a prevalence of about 5.27% occurring in this population. Compared to pregnant women with an adverse pregnancy and childbirth history, pregnant women without an adverse pregnancy and childbirth history were more likely to suffer from GDM, with a prevalence of about 4.35% occurring in this population.

### 3.2. Air Pollution Exposure

The statistical description and Spearman’s correlation analysis of the average exposure levels of the pollutants in different exposure windows for the pregnant women are shown in Figure S1 and Table S1, respectively. The medians of the PM_10_, PM_2.5_, SO_2_, NO_2_, CO, and O_3_ in the study periods were 119.79–125.30 μg/m^3^, 59.34–68.71 μg/m^3^, 21.11–23.31 μg/m^3^, 37.12–39.79 μg/m^3^, 1.20–1.28 mg/m^3^, and 102.22–123.86 μg/m^3^, respectively. According to the upper and lower quartiles, the group exposure levels for each pollutant within each window were quite different. Comparing the exposure levels of the same pollutant in each window, it could be seen that the exposure levels of the same pollutant in different windows were approximately the same. According to the Spearman’s correlation coefficient, the correlations between the pollutants in each window were strong, so it was not possible to include any two pollutants directly and simultaneously in the logistic regression model.

### 3.3. Association between Air Pollution and the Risk of GDM

The ORs and 95% CIs of each pollutant for GDM in the single-pollutant model are shown in Table 2. In the model without an adjustment for the covariates, each 10 μg/m^3^ increase in NO_2_ exposure in the T and T2 periods had a significant effect on the risk of GDM, with OR values of 1.144 (95% CI: 1.020–1.283) and 1.128 (95% CI: 1.034–1.231), respectively. In the model adjusted for the covariates, each 20 μg/m^3^ increase in PM_10_ exposure during the Pre_T period increased the risk of GDM by 13.8% (OR = 1.138, 95% CI: 1.041–1.246). Each 10 μg/m^3^ increase in NO_2_ exposure during the T and T2 periods also significantly increased the risk of GDM, with OR values of 1.305 (95% CI: 1.088–1.566) and 1.296 (95% CI: 1.120–1.500), respectively.

Table 3 shows the ORs and 95% CIs of the effect of each pollutant on GDM in the multi-pollutant model. No significant effect of any pollutant on GDM was found in the model without an adjustment for the covariates. In the model adjusted for the covariates, each 20 μg/m^3^ increase in PM_2.5_ in the Pre_T, T, and T1 periods increased the risk of GDM by 1.6% (OR = 1.016, 95% CI: 1.001–1.028), 2.1% (OR = 1.021, 95% CI: 1.004–1.038), and 1.5% (OR = 1.015, 95% CI: 1.006–1.024), respectively. Each 20 μg/m^3^ increase in PM_10_ exposure in the Pre_T, T, and T2 periods also increased the risk of GDM, with OR values of 1.011 (95% CI: 1.001–1.022), 1.015 (95% CI: 1.005–1.026), and 1.016 (95% CI: 1.005–1.027), respectively.

### 3.4. Stratified Analyses

In both the single-pollutant model and multi-pollutant model, SO_2_, CO, and O_3_ had no significant effect on the risk of GDM after an adjustment for the confounders, so only the results for the other three pollutants are presented in the stratified analysis.

The effect of air pollution on the risk of GDM in pregnant women of different ages is shown in Figure S2. The results of the single-pollutant model showed that, for pregnant women aged 30–35 years, each 10 μg/m^3^ increase in NO_2_ exposure in the T and T2 periods significantly increased the risk of GDM, with ORs of 1.710 (95% CI: 1.243–1.352) and 1.595 (95% CI: 1.228–2.072), respectively. However, this finding was not found in the multi-pollutant model. For nulliparous women, the multi-pollutant model showed that, for each 20 μg/m^3^ increase in PM_2.5_ during the T1 period, the risk of GDM increased by 1.6% (OR = 1.016, 95% CI: 1.002–1.030) (Figure S3). Figure S4 shows the effect of GDM risk in women with different previous pregnancy and childbirth. Pregnant women with previous adverse pregnancy and childbirth were more sensitive to PM_10_ exposure in the T and T2 periods, whereas those without were more sensitive to NO_2_ exposure in the T and T2 periods. For pregnant women with less than a bachelor’s education, the single-pollutant model showed that, for each 20 μg/m^3^ increase in PM_10_ during the T2 period, the risk of GDM was increased by 19.0% (OR = 1.190, 95% CI: 1.029–1.376) (Figure S5). Each 10 μg/m^3^ increase in NO_2_ exposure during the T and T2 periods also had a significant effect on the risk of GDM, with ORs of 1.470 (95% CI: 1.135–1.903) and 1.473 (95% CI: 1.194–1.818), respectively (Figure S5).

## 4. Discussion

As a heavily polluted city with a large population, the data from Handan are representative and universal. This study investigated the effect of air pollution exposure during preconception and pregnancy on the risk of GDM and the sensitivity of different populations using data from pregnant women who gave birth at the Handan Maternal and Child Health Hospital. The results showed that exposure to PM_2.5_, PM_10_, and NO_2_ had adverse effects on the risk of GDM in pregnant women, and this effect varied across populations.

The prevalence of GDM has increased significantly in recent decades. However, about half of patients do not have classic risk factors [42]. Over the 60-year period from 1950 to 2015, the emissions of air pollutants showed a clear upward trend in all regions of Asia [43]. The available evidence suggests that environmental pollutants such as air pollution may be risk factors for glucose tolerance and glucose homeostasis in normal women [19]. In our study, the single-pollutant model adjusted for covariates showed that NO_2_ exposure in the T and T2 periods significantly increased the risk of GDM in pregnant women. Several studies have also reported a significant association between exposure to air pollution and an increased risk of GDM. Jo et al. found that exposure to NO_2_ per 10.4 ppb increment, PM_2.5_ per 6.5 μg/m^3^ increment, and PM_10_ per 16.1 μg/m^3^ increment during the Pre_T period was associated with an increased risk of GDM [44]. Shen et al. showed that exposure to PM_2.5_ and SO_2_ during the T1 and T2 periods significantly increased the risk of GDM [22]. After controlling for nine covariates, Hu et al. found that the odds of GDM increased by 16%, 15%, 9%, and 12% for each 5 μg/m^3^ increase in PM_2.5_ and 5 ppb increase in O_3_ during the T1 and T2 periods, respectively [35]. There are also some studies that have reported opposite results, which may be related to differences in the exposure assessment methods, populations, geographical locations, and adjusted covariates [21,45]. The results of this study also confirmed the adverse effects of preconception exposure to air pollution on GDM. In the multi-pollutant model adjusted for other pollutants and the covariates, PM_2.5_ and PM_10_ exposure in the Pre_T period significantly increased the risk of GDM. Several previous studies have also examined preconception exposure and found that air pollution during this period may increase maternal systemic oxidative stress and inflammation [22,44,45,46,47]. This suggests that preconception is also a critical exposure window and that preconception care focusing on lifestyle modification and a reduction in adverse risk factors may be effective in preventing pregnancy complications.

In our study, measurements from monitoring stations were directly used as a metric for the exposure of the pregnant women to air pollution due to the lack of detailed data on their activity levels. However, complex human activities may have influenced the time and space of the exposure and thus the level of exposure. A survey of human activity patterns showed that people spend up to 86.9% of their total time indoors [48]. Buildings and indoor anthropogenic factors (e.g., air purifiers) may shield people from some pollutants from outdoor sources, while some indoor emission sources (e.g., cooking and furniture) expose people to additional pollutants [49]. There is growing evidence of differences in indoor PM and NOx exposure between income groups in developed countries [50]. In addition, certain studies have shown that there are small seasonal differences between indoor and outdoor pollutant concentrations [51]. Human exposure to air pollutants results from a combination of outdoor and indoor sources. The composition of indoor and outdoor pollution may be the same, and the exposure–response relationship is not affected by the source of a particular pollutant [52]. However, when pollutants have both indoor and outdoor sources, ignoring the moderating effect of indoor factors on human exposure may lead to a misclassification of this exposure. Therefore, we recommend that future studies consider indoor factors in their exposure assessments whenever possible.

The effect of PM_10_ exposure in the Pre_T period on GDM in the multi-pollutant model (OR = 1.011, 95% CI: 1.001–1.022) was lower than that in the single-pollutant model (OR = 1.138, 95% CI: 1.041–1.246), indicating that co-exposure to other pollutants in the Pre_T period was also a risk factor for GDM. Several foreign studies have also shown that the effects of pollutants in single-pollutant models are different from those in two-pollutant models. After an adjustment for PM_2.5_ and O_3_, Jo et al. found that the ORs of NO_2_ for GDM decreased from 1.10 (95% CI: 1.07–1.13) to 1.09 (95% CI: 1.05–1.13) and 1.04 (95% CI: 1.00–1.08), respectively [44]. In the study by Pan et al., the OR of CO for GDM decreased from 1.08 (95% CI: 1.00–1.15) to 1.05 (95% CI: 0.98–1.13) after an adjustment for O_3_ [23]. The results of Choe et al. showed that the OR of PM_2.5_ exposure on GDM in pregnant women during the T2 period was 1.08 (95% CI: 1.00–1.15), and the OR was 1.07 (95% CI: 1.00–1.15) after an adjustment for proximity to roads [53]. Different air pollutants coexist in the real environment, and there may be synergistic/antagonistic effects of different air pollutants on GDM in pregnant women. Therefore, it is necessary to adjust for the effects of other air pollutants besides the target ones. Due to the strong correlation between the pollutants, although previous studies have adjusted for pollutants with relatively weak correlations with the studied pollutants, there were still multicollinearity problems between the independent variables in the model, which may have affected the accuracy of the parameter estimation.

To improve the estimation of parameters under the condition of multicollinearity, researchers have developed various statistical analysis methods, such as principal component regression, partial least squares regression, and ridge regression [25,26,27]. A principal component analysis is a multivariate statistical analysis method that uses orthogonal transformations to transform a set of possibly correlated variables into a set of linearly uncorrelated variables (principal components). As an easy to apply and efficient method (most of the variability in the original exposure is retained by a few factors), it has been widely used in multi-pollutant modelling [54,55,56]. Several recent studies have provided new ideas for multi-pollutant modelling, such as Bayesian kernel machine regression (BKMR) [57] and profile regression [58]. In the future, there is a need to explore more appropriate methods to solve the multicollinearity problem in multi-pollutant models, in order to accurately reflect the effects of pollutants.

In the subgroup analysis, pregnant women aged 30–35 years, nulliparous women, and those with less than a bachelor’s education were sensitive groups, which is consistent with previous studies [24,59]. The effect of age on the association between air pollution and GDM is currently controversial. As the body’s ability to metabolize lipids declines with age, older pregnant women are more likely to develop atherosclerosis and cardiovascular disease, which may be induced or exacerbated by air pollution [60]. It has also been suggested that younger pregnant women may be more susceptible to the effects of air pollution due to their higher respiratory minute volumes, higher levels of activity, and more time spent outdoors [61]. Several studies have suggested that first pregnancy is also a significant risk factor for GDM. For nulliparous women, the abnormal immune response to the initial exposure of fetal-derived villi leads to GDM, and maternal exposure to air pollution may aggravate this immune response [62,63]. Pregnant women with lower levels of education have a correspondingly lower social status and a longer exposure to outdoor air pollution during pregnancy. At the same time, their potential lack of knowledge about perinatal health care and protection makes them a high-risk group [64]. In addition, higher education is, to some extent, related to economic status, which may affect the basic health and nutritional status of pregnant women.

This research has several strengths. It is the first study to focus on the residents of small- and medium-sized cities. In the case of Handan city, the health effects of air pollution on pregnant women with GDM were discussed. Another advantage is that the effects of air pollutants were evaluated after adjusting for other pollutants, and the principal component logistic regression model was cleverly used to solve the collinearity problem among the pollutants included in the model, making the results closer to reality. At the same time, there were some shortcomings in this study. Due to the lack of information in the original data, it was not possible to adjust for some potentially important confounding variables, including maternal body mass index, maternal alcohol consumption, maternal smoking status, maternal stress, and noise exposure, etc. Secondly, due to the limitations of the data obtained, there was no information on the mobility of the pregnant women during their pregnancy, the exposure of the pregnant women in their homes or workplaces could not be taken into account, and there may have been an exposure misclassification, but the exposure misclassification would not be differential.

## 5. Conclusions

This study provided evidence of an association between air pollution and the prevalence of GDM. Exposure to PM_2.5_, PM_10_, and NO_2_ during preconception and pregnancy has adverse effects on the risk of GDM. As a result, pregnant women need to pay attention to air pollution protection not only during pregnancy, but also during preconception. Advanced age, non-delivery, and low educational level are high risk factors for the risk of air pollution on GDM in pregnant women. Therefore, susceptible individuals should be more aware of the dangers of air pollution and try to avoid outdoor activities in weather with a poor air quality. In addition, this study confirmed that the results of the multi-pollutant model were closer to the real situation than those of the single-pollutant model. However, due to the correlation between the pollutants, it is necessary to explore suitable methods to solve the multicollinearity problem in the future.

## Figures and Tables

**Table 1:** Characteristics of study population.

Characteristics	Total *n* (%)	GDM *n* (%)	Non-GDM *n* (%)	*p* ^a^
Total	9820 (100.00)	372 (3.79)	9448 (96.21)	
Age				0.002
≤25	937 (9.54)	42 (4.48)	895 (95.52)	
(25, 30)	3796 (38.66)	120 (3.16)	3676 (96.84)	
(30, 35)	3494 (35.58)	126 (3.61)	3368 (96.39)	
>35	1593 (16.22)	84 (5.27)	1509 (94.73)	
Education				0.620
Less than bachelor	4773 (48.60)	186 (3.9)	4587 (96.1)	
Bachelor or above	5047 (51.40)	186 (3.69)	4861 (96.31)	
Health insurance				0.108
Urban and rural medical insurance	4126 (42.02)	176 (4.27)	3950 (95.73)	
Employee medical insurance	3610 (36.76)	124 (3.43)	3486 (96.57)	
None	2084 (21.22)	72 (3.45)	2012 (96.55)	
Parity				0.464
Nulliparous	4714 (48.00)	186 (3.95)	4528 (96.05)	
Multiparous	5106 (52.00)	186 (3.64)	4920 (96.36)	
Conception year				0.202
2017	1124 (11.45)	55 (4.89)	1069 (95.11)	
2018	4221 (42.98)	129 (3.06)	4092 (96.94)	
2019	4475 (45.57)	188 (4.20)	4287 (95.80)	
Conception season				0.952
Spring	2271 (23.13)	84 (3.70)	2187 (96.30)	
Summer	2877 (29.30)	114 (3.96)	2763 (96.04)	
Autumn	2629 (26.77)	98 (3.73)	2531 (96.27)	
Winter	2043 (20.80)	76 (3.72)	1967 (96.28)	
Previous adverse pregnancy and childbirth				<0.001
No	6827 (69.52)	297 (4.35)	6530 (95.65)	
Yes	2993 (30.48)	75 (2.51)	2918 (97.49)	

^a^ Analysis of χ^2^ test.

**Table 2:** Effect of each pollutant in single-pollutant model.

Pollutants	Periods	OR (95% CI) ^a^	OR (95% CI) ^b^
PM_2.5_	Pre_T	1.018 (0.940, 1.103)	1.110 (0.959, 1.283)
	T1	0.998 (0.921, 1.081)	1.019 (0.867, 1.197)
	T2	1.055 (0.975, 1.141)	1.051 (0.911, 1.214)
	T	1.053 (0.942, 1.177)	1.082 (0.865, 1.352)
PM_10_	Pre_T	1.045 (0.990, 1.103)	**1.138 (1.041, 1.246)** *
	T1	0.999 (0.946, 1.056)	1.038 (0.939, 1.148)
	T2	1.030 (0.974, 1.090)	1.070 (0.968, 1.183)
	T	1.026 (0.951, 1.107)	1.097 (0.960, 1.253)
SO_2_	Pre_T	0.974 (0.890, 1.067)	1.025 (0.911, 1.153)
	T1	0.952 (0.868, 1.044)	0.978 (0.863, 1.109)
	T2	1.088 (0.991, 1.193)	1.143 (0.974, 1.288)
	T	1.021 (0.912, 1.144)	1.096 (0.939, 1.279)
NO_2_	Pre_T	1.012 (0.929, 1.101)	1.094 (0.952, 1.257)
	T1	1.033 (0.949, 1.123)	1.092 (0.940, 1.268)
	T2	**1.128 (1.034, 1.231)** *	**1.296 (1.120, 1.500)** *
	T	**1.144 (1.020, 1.283)** *	**1.305 (1.088, 1.566)** *
CO	Pre_T	0.996 (0.894, 1.110)	1.137 (0.979, 1.320)
	T1	0.988 (0.885, 1.102)	1.058 (0.893, 1.254)
	T2	1.116 (0.999, 1.248)	1.151 (0.989, 1.339)
	T	1.078 (0.938, 1.239)	1.166 (0.961, 1.414)
O_3_	Pre_T	1.015 (0.968, 1.064)	0.971 (0.873, 1.081)
	T1	1.002 (0.956, 1.050)	1.039 (0.932, 1.159)
	T2	0.966 (0.922, 1.012)	0.968 (0.870, 1.077)
	T	0.969 (0.909, 1.034)	1.004 (0.867, 1.163)

^a^ Crude models. ^b^ Models were additional adjusted for maternal age, education, health insurance, parity, conception year, conception season, and previous adverse pregnancy and childbirth. * *p* < 0.05. Bold indicates that the pollutant is a risk factor.

**Table 3:** Effect of each pollutant in multi-pollutant model.

Pollutants	Periods	OR (95% CI) ^a^	OR (95% CI) ^b^
PM_2.5_	Pre_T	0.974 (0.940, 1.009)	**1.016 (1.003, 1.028)** *
	T1	0.997 (0.959, 1.037)	**1.015 (1.006, 1.024)** *
	T2	1.027 (0.985, 1.071)	1.014 (0.999, 1.030)
	T	1.005 (0.961, 1.050)	**1.021 (1.004, 1.038)** *
PM_10_	Pre_T	0.973 (0.942, 1.006)	**1.011 (1.001, 1.022)** *
	T1	0.995 (0.964, 1.028)	1.009 (0.999, 1.018)
	T2	1.014 (0.991, 1.037)	**1.016 (1.005, 1.027)** *
	T	1.005 (0.968, 1.044)	**1.015 (1.005, 1.026)** *
SO_2_	Pre_T	1.036 (0.987, 1.088)	0.938 (0.917, 0.960) *
	T1	1.028 (0.982, 1.076)	0.990 (0.964, 1.016)
	T2	0.999 (0.950, 1.052)	0.969 (0.939, 0.999) *
	T	1.032 (0.974, 1.092)	0.975 (0.942, 1.009)
NO_2_	Pre_T	1.032 (0.975, 1.091)	1.011 (0.998, 1.025)
	T1	1.003 (0.951, 1.057)	0.998 (0.962, 1.035)
	T2	0.945 (0.886, 1.007)	1.001 (0.987, 1.015)
	T	0.985 (0.936, 1.036)	1.002 (0.969, 1.037)
CO	Pre_T	1.025 (0.990, 1.062)	0.968 (0.950, 0.987) *
	T1	1.023 (0.988, 1.060)	0.995 (0.974, 1.016)
	T2	0.997 (0.974, 1.021)	1.000 (0.981, 1.020)
	T	1.001 (0.922, 1.087)	0.992 (0.964, 1.020)
O_3_	Pre_T	0.993 (0.973, 1.014)	0.989 (0.982, 0.996) *
	T1	1.001 (0.983, 1.020)	1.000 (0.990, 1.011)
	T2	1.010 (0.995, 1.025)	1.002 (0.992, 1.011)
	T	1.010 (0.989, 1.031)	1.004 (0.986, 1.021)

^a^ Models were adjusted for all other pollutants. ^b^ Models were additional adjusted for maternal age, education, health insurance, parity, conception year, conception season, and previous adverse pregnancy and childbirth. * *p* < 0.05. Bold indicates that the pollutant is a risk factor.

## Data Availability

The datasets generated during and/or analyzed during the current study are not publicly available but are available from the corresponding author on reasonable request.

## Supplementary Materials

The following supporting information can be downloaded at: https://www.mdpi.com/article/10.3390/toxics11090728/s1, Figure S1: Distributions of average exposure levels of pollutants during different periods. The bottom bar, the horizontal line, and the top bar of the box indicates the 25th, 50th, and 75th percentiles, respectively. The box whiskers present the 5th and 95th percentiles.; Figure S2: Effects of air pollutants on the risk of GDM in pregnant women of different ages in single-pollutant models (a) and multi-pollutant models (b). All models were adjusted for maternal education, health insurance, parity, conception year, conception season, and previous adverse pregnancy and childbirth. * *p* < 0.05; Figure S3: Effects of air pollutants on the risk of GDM in pregnant women of different parity in single-pollutant models (a) and multi-pollutant models (b). All models were adjusted for maternal age, education, health insurance, conception year, conception season, and previous adverse pregnancy and childbirth. * *p* < 0.05; Figure S4: Effects of air pollutants on the risk of GDM in pregnant women with different previous pregnancy and childbirth in single-pollutant models (a) and multi-pollutant models (b). All models were adjusted for maternal age, education, health insurance, parity, conception year and conception season. * *p* < 0.05; Figure S5: Effects of air pollutants on the risk of GDM in pregnant women with different education levels in single-pollutant models (a) and multi-pollutant models (b). All models were adjusted for maternal age, health insurance, parity, conception year, conception season, and previous adverse pregnancy and childbirth. * *p* < 0.05. Table S1: Spearman’s correlations of the average exposure levels of pollutants during different periods; Table S2: Principal component analysis results of six pollutants; Table S3: Principal component analysis results of six pollutants and confounding variables.
