# Supplementary material for: Effects of Air Pollution Exposure during Preconception and Pregnancy on Gestational Diabetes Mellitus
Source: Toxics. 2023 Aug 24;11(9):728. doi: 10.3390/toxics11090728 (PMC10534707; doi:10.3390/toxics11090728)
Supplement: Supplementary file 1 [file toxics-11-00728-s001.zip › toxics-2549952-supplementary.pdf]

Supplementary Material

Part 1 Methods for building logistic regression models

Let the dependent variable y be a dichotomous variable that takes the value 1 (diseased) or 0 (not diseased), $X_1, X_2, \ldots, X_m$ are the m independent variables that affect the value of y, $P = P(y=1 \mid X_1, X_2, \cdots, X_m)$ is the conditional probability of positive results under the effect of m independent variables (exposure factors). The logistic regression model can be expressed as follows:

$$\text{logit}(P) = \ln\left(P/1-P\right) = \beta_0 + \beta_1 X_1 + \beta_2 X_2 + \ldots + \beta_m X_m \quad (1.1)$$

Where β_0 is the natural log of the ratio of disease probability to non-disease probability when all exposure factors are 0. If X_i is a continuous variable, $\beta_i (i = 1, 2, \cdots, m)$ represents the average change of $\text{logit}(P)$ when the independent variable X_i changes by one unit under the condition that other independent variables are fixed. If X_i is a categorical variable, β_i represents the natural log of the odds ratio of the prevalence in the exposed group versus the unexposed group, given that the other independent variables are fixed.

If risk factor X_i is a continuous variable, the OR and 95% CI caused by an increase of c units are calculated as follows:

$$\text{OR}_i = \exp(\beta_i \times c) \quad (1.2)$$

$$\text{CI}_i = \exp[(\beta_i \pm 1.96 \times \text{SE}_i) \times c] \quad (1.3)$$

Where, SE_i is the standard deviation of β_i.

If risk factor X_i is a categorical variable, the OR and 95% CI of the exposed group and the non-exposed group are calculated as follows:

$$\text{OR}_i = \exp(\beta_i) \quad (1.4)$$

$$\text{CI}_i = \exp(\beta_i \pm 1.96 \times \text{SE}_i) \quad (1.5)$$

When $\beta = 0$, $\text{OR} = 1$, means that factor X_i does not play a role in the occurrence of the disease. When $\beta < 0$, $\text{OR} < 1$, represents X_i is a risk factor. When $\beta > 0$, $\text{OR} > 1$, indicates X_i is a protective factor.

Part 2 Methods for building principal component logistic regression models

First, the original independent variable X of the model was normalized into X^*, and then principal component analysis is carried out on the standardized independent variables. The first r principal components are selected according to the cumulative contribution rate of characteristic roots (≥ 90%), and the load matrix $\boldsymbol{\varphi}$ is divided into two parts:

$$\boldsymbol{\varphi} = (\boldsymbol{\varphi}_1 / \boldsymbol{\varphi}_2) \tag{2.1}$$

The score values of the first r principal components are calculated as follows:

$$Z = X^* \boldsymbol{\varphi}_1 \tag{2.2}$$

The score values of the first r principal components are used for logistic regression analysis, and the estimated regression coefficient α and its covariance matrix Σ are obtained. Then, the standardized original variable is substituted back into the equation, and the regression coefficient β^* and its standard deviation SE^* of the standardized original variable are obtained as follows:

$$\beta^* = \boldsymbol{\varphi}_1 \alpha \tag{2.3}$$

$$\mathrm{SE}^* = \sqrt{\mathrm{diag}(\boldsymbol{\varphi}_1 \Sigma \boldsymbol{\varphi}_1^{\mathrm{T}})} \tag{2.4}$$

Where $\mathrm{diag}(\boldsymbol{\varphi}_1 \Sigma \boldsymbol{\varphi}_1^{\mathrm{T}})$ is the diagonal element of the matrix $\boldsymbol{\varphi}_1 \Sigma \boldsymbol{\varphi}_1^{\mathrm{T}}$.

The regression coefficient β and its standard deviation SE of the original variable are calculated as follows:

$$\beta = \beta^* / S_X \tag{2.5}$$

$$\mathrm{SE} = \mathrm{SE}^* / S_X \tag{2.6}$$

Where S_X is the standard deviation of the original variable.

The results of standardized principal component analysis of the six pollutants were shown in Table S2. The accumulated contribution rate of the first three principal components in each exposure window was more than 90%, indicating that the most information of the six pollutants in each exposure window was presented.

The results of standardized principal component analysis of the six pollutants and seven confounding variables were shown in Table S3. According to the results, the first eight principal components contained most information, so the first eight principal components were used to build logistic regression models.

Part 3 Tables

Table S1 Spearman's correlations[a] of the average exposure levels of pollutants during different periods.

Periods	Pollutants	$PM_{2.5}$	PM_{10}	SO_2	NO_2	CO	O_3
Pre_T	$PM_{2.5}$	1.00	0.93	0.70	0.80	0.79	-0.84
	PM_{10}		1.00	0.68	0.80	0.74	-0.78
	SO_2			1.00	0.68	0.76	-0.66
	NO_2				1.00	0.77	-0.84
	CO					1.00	-0.71
	O_3						1.00
T1	$PM_{2.5}$	1.00	0.94	0.68	0.79	0.80	-0.83
	PM_{10}		1.00	0.67	0.80	0.75	-0.80
	SO_2			1.00	0.60	0.73	-0.57
	NO_2				1.00	0.75	-0.87
	CO					1.00	-0.70
	O_3						1.00
T2	$PM_{2.5}$	1.00	0.93	0.54	0.76	0.80	-0.85
	PM_{10}		1.00	0.57	0.80	0.76	-0.79
	SO_2			1.00	0.52	0.64	-0.39
	NO_2				1.00	0.74	-0.82
	CO					1.00	-0.72
	O_3						1.00
T	$PM_{2.5}$	1.00	0.94	0.58	0.80	0.75	-0.83
	PM_{10}		1.00	0.57	0.80	0.71	-0.73
	SO_2			1.00	0.48	0.70	-0.33
	NO_2				1.00	0.74	-0.79
	CO					1.00	-0.61
	O_3						1.00

[a] All correlations are statistically significant ($p < 0.001$).

Abbreviations: Pre_T, preconception; T1, first trimester; T2, second trimester; T, the first two trimesters.

Table S2 Principal component analysis results of six pollutants.

Principal component	Contribution rate				Accumulated contribution rate			
	Pre_T	T1	T2	T	Pre_T	T1	T2	T
D1	0.80	0.79	0.76	0.74	0.80	0.79	0.76	0.74
D2	0.08	0.10	0.12	0.13	0.89	0.89	0.88	0.87
D3	0.05	0.05	0.05	0.05	0.93	0.94	0.92	0.92
D4	0.03	0.03	0.04	0.04	0.97	0.97	0.96	0.96
D5	0.03	0.02	0.03	0.03	0.99	0.99	0.99	0.99
D6	0.01	0.01	0.01	0.01	1.00	1.00	1.00	1.00

Abbreviations: Pre_T, preconception; T1, first trimester; T2, second trimester; T, the first two trimesters.

Table S3 Principal component analysis results of six pollutants and confounding variables.

Principal component	Contribution rate				Accumulated contribution rate			
	Pre_T	T1	T2	T	Pre_T	T1	T2	T
D1	0.39	0.43	0.38	0.39	0.39	0.43	0.38	0.39
D2	0.11	0.11	0.12	0.11	0.50	0.54	0.49	0.51
D3	0.11	0.11	0.11	0.11	0.61	0.65	0.60	0.62
D4	0.10	0.09	0.10	0.09	0.70	0.74	0.71	0.71
D5	0.08	0.07	0.07	0.08	0.78	0.81	0.77	0.79
D6	0.06	0.05	0.06	0.05	0.84	0.86	0.84	0.84
D7	0.05	0.04	0.05	0.04	0.89	0.90	0.88	0.88
D8	0.04	0.03	0.04	0.04	0.93	0.93	0.92	0.92
D9	0.03	0.03	0.03	0.03	0.96	0.96	0.95	0.95
D10	0.01	0.02	0.02	0.02	0.97	0.98	0.97	0.97
D11	0.01	0.01	0.02	0.02	0.99	0.99	0.99	0.99
D12	0.01	0.01	0.01	0.01	1.00	1.00	1.00	1.00
D13	0.00	0.00	0.00	0.00	1.00	1.00	1.00	1.00

Abbreviations: Pre_T, preconception; T1, first trimester; T2, second trimester; T, the first two trimesters.

Part 4 Figures

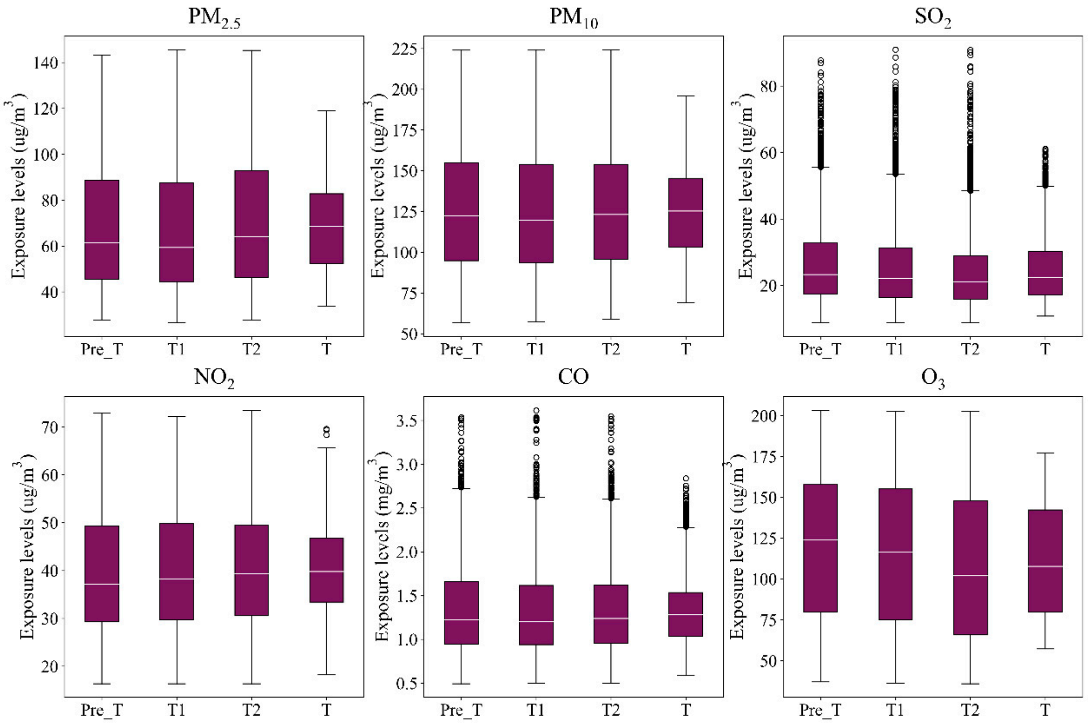

Figure S1. Distributions of average exposure levels of pollutants during different periods. The bottom bar, the horizontal line, and the top bar of the box indicates the 25th, 50th, and 75th percentiles, respectively. The box whiskers present the 5th and 95th percentiles.

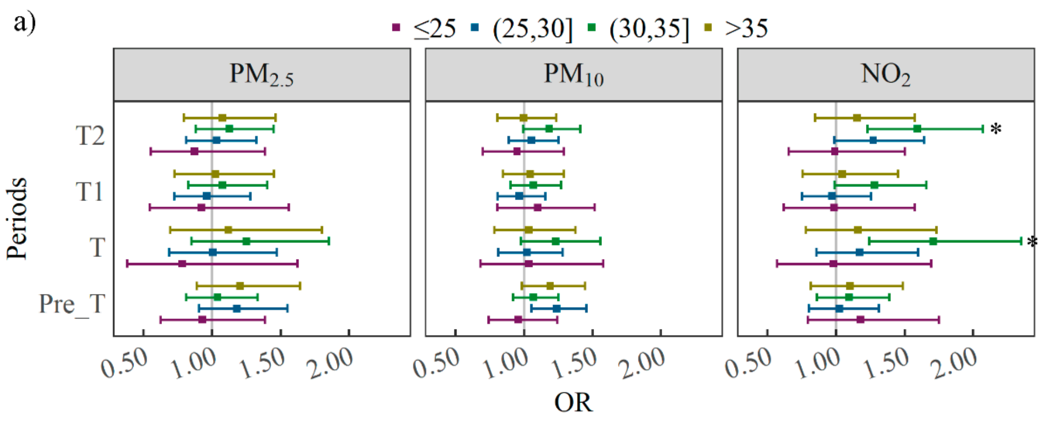

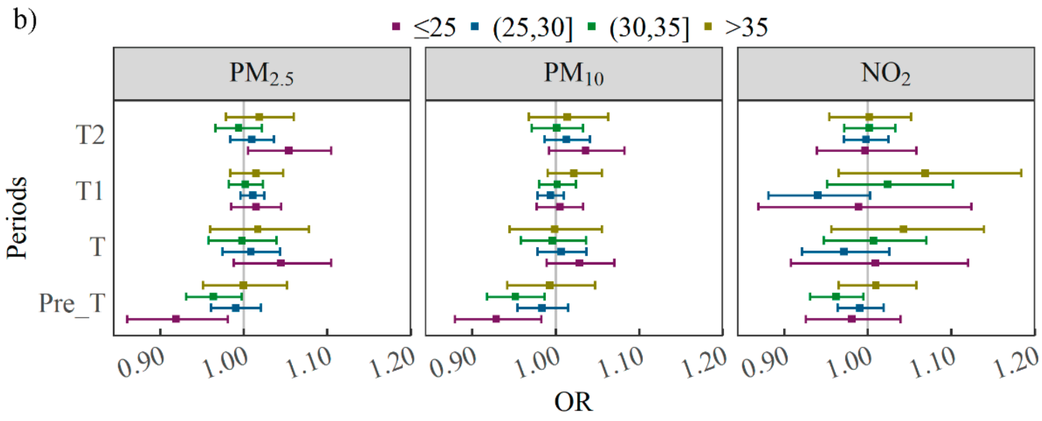

Figure S2. Effects of air pollutants on the risk of GDM in pregnant women of different ages in single-pollutant models (a) and multi-pollutant models (b). All models were adjusted for maternal education, health insurance, parity, conception year, conception season, and previous adverse pregnancy and childbirth. * $P < 0.05$.

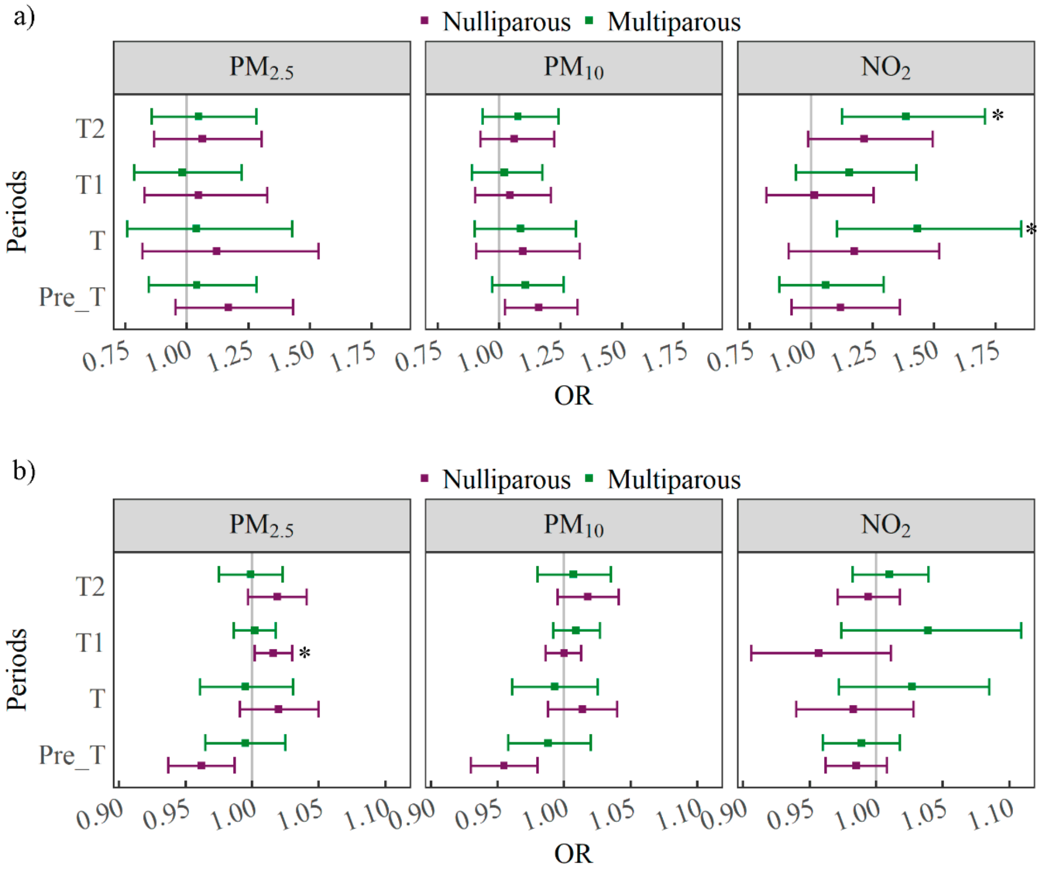

Figure S3. Effects of air pollutants on the risk of GDM in pregnant women of different parity in single-pollutant models (**a**) and multi-pollutant models (**b**). All models were adjusted for maternal age, education, health insurance, conception year, conception

season, and previous adverse pregnancy and childbirth. * P <0.05.

a)

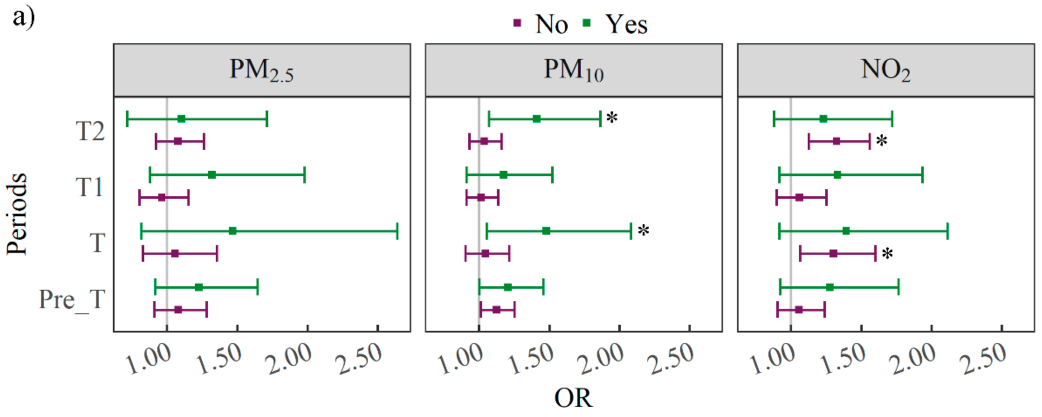

b)

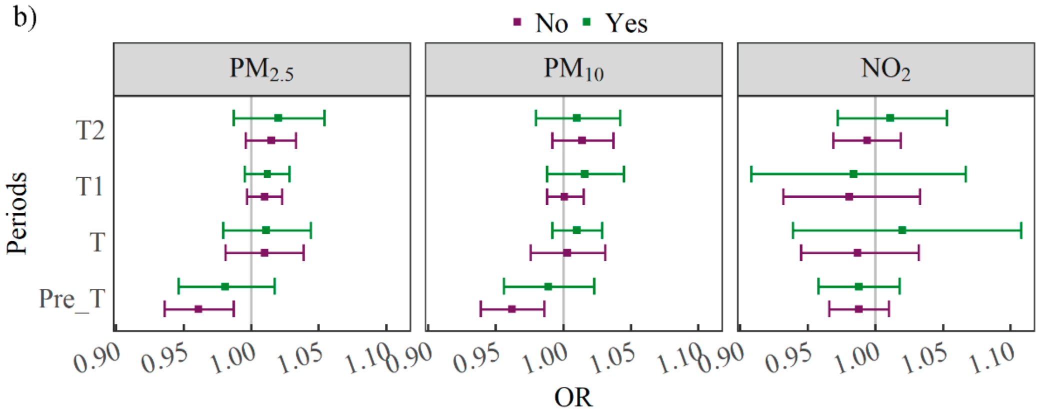

Figure S4. Effects of air pollutants on the risk of GDM in pregnant women with different previous pregnancy and childbirth in single-pollutant models (**a**) and multi-pollutant models (**b**). All models were adjusted for maternal age, education, health insurance, parity, conception year and conception season. * P <0.05.

a)

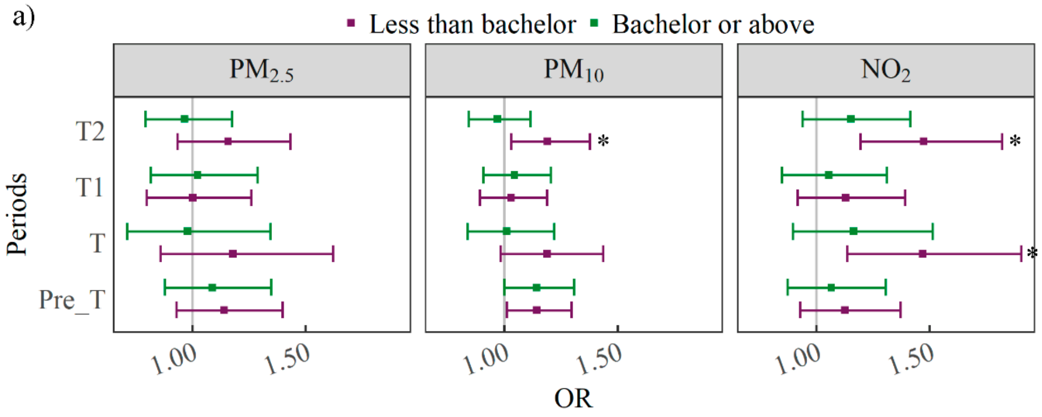

b)

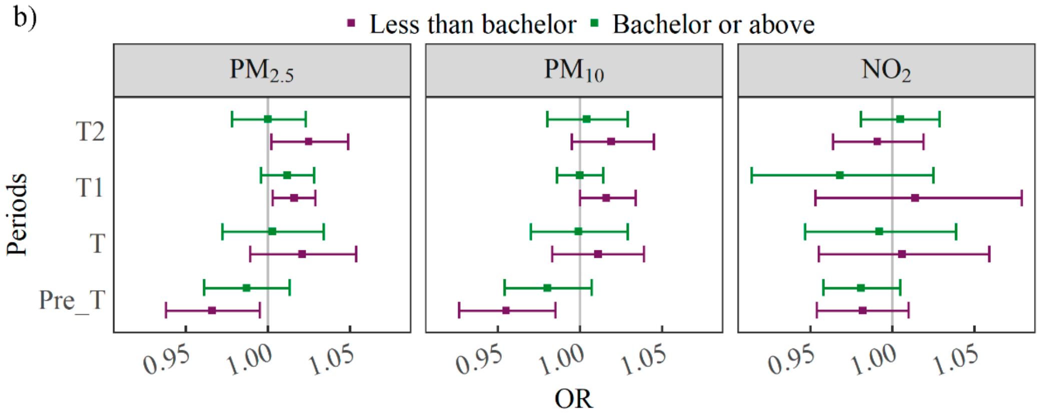

Figure S5. Effects of air pollutants on the risk of GDM in pregnant women with different education levels in single-pollutant models (**a**) and multi-pollutant models (**b**). All models were adjusted for maternal age, health insurance, parity, conception year, conception season, and previous adverse pregnancy and childbirth. * $P < 0.05$.
